# Enhancement of Therapeutic Potential of Oncolytic Virus with Homologous Tumor Cell Membranes for Pancreatic Cancer

**DOI:** 10.1049/2024/9970665

**Published:** 2024-02-13

**Authors:** Wei Chen, Hui Liu, Yue Chen, Meng Gao

**Affiliations:** ^1^Department of Gastroenterology, The Second People's Hospital of Hefei, Hefei Hospital Affiliated to Anhui Medical University, Hefei, 230011, China; ^2^Department of Gastroenterology, The Second People's Hospital of Hefei Affiliated to Bengbu Medical College, Hefei 230011, China; ^3^Department of Gastroenterology, The Second People's Hospital of Hefei, Hefei 230011, China; ^4^School of Clinical Medicine, Anhui Medical University, Hefei 230011, China

## Abstract

Pancreatic cancer is a leading cause of cancer-related deaths worldwide. Conventional therapies often provide limited success, necessitating the need for novel therapeutic strategies. Oncolytic viruses (OVs) are a class of viruses that specifically target and kill cancer cells while leaving normal cells unharmed. These viruses have shown promise in the treatment of various cancers, including pancreatic cancer. However, their use in clinical settings has been limited by several factors. Their inability to efficiently infect and kill tumor cells. To overcome this limitation, a cell membrane-coated oncolytic virus was developed. However, the necessity of homologous and nonhomologous tumor cell membranes for their function has not yet been proven. This novel virus displayed increased infectivity and killing activity against tumor cells compared to nonhomologous tumor cell membranes and noncoated viruses. We believe that the homologous tumor cell membranes-coated OVs can enhance the therapeutic potential for pancreatic cancer therapy.

## 1. Introduction

Pancreatic cancer remains a significant health care challenge with a high rate of mortality worldwide [1–4]. Conventional treatment methods, such as surgery, radiotherapy, and chemotherapy, have shown limited efficacy and significant side effects. Therefore, the search for novel and more effective treatment strategies is crucial. Oncolytic viruses (OVs), which are viruses capable of specifically replicating in and killing tumor cells while sparing normal cells, have emerged as a potential new approach for the treatment of pancreatic cancer [5–7]. However, these viruses also face a number of challenges.

OVs have shown great potential in targeting and killing pancreatic cancer cells. This is due to the unique biological characteristics of tumor cells that render them susceptible to virus infection [8, 9]. For example, some pancreatic cancer cells express high levels of epidermal growth factor receptor (EGFR), which can be targeted by some OVs. By binding to EGFR, these viruses are able to specifically infect and kill pancreatic cancer cells [10, 11]. However, the use of OVs in pancreatic cancer treatment is also facing a number of challenges. First, not all pancreatic cancer cells can be targeted by these viruses, limiting their therapeutic effect [12]. Second, the stability of OVs in the body is also a concern [13]. Finally, the effective delivery of these viruses to the tumor site remains a challenge due to potential clearance by the immune system or limited penetration into the tumor [14–17]. The systemic delivery of OVs remains a major challenge in clinical treatment. The viruses are easily recognized by the body's immune system and eliminated through antibodies or other mechanisms, resulting in low pharmaceutical value and insufficient therapeutic effect [18–22]. Despite these challenges, the use of OVs offers a promising new approach for the treatment of pancreatic cancer. With further research and development, it is hoped that these viruses can be improved to enhance their therapeutic effect while addressing the issues of stability and delivery.

To address this issue, tumor cell membranes can be used to modify OVs to mask their viral characteristics and reduce their immunogenicity, thereby increasing their survival time in the body and improving their therapeutic effect. This modification alters the virus's immunological properties while maintaining its oncolytic activity, allowing the virus to evade immune clearance and target tumors more efficiently. In the field of nanomedicine, surface modification of nanoparticles with tumor cell membranes can help improve their targeting ability toward tumors and enhance the therapeutic effect of their nanodrugs [23–26]. In addition, the use of secretion-based exosomes has also been commonly employed to address delivery-related challenges, providing a potential solution for targeted delivery within the body. This approach, to some extent, can help overcome targeting issues in vivo [27]. Genetic engineering techniques have become an effective means of regulating extracellular vesicles and enhancing oncolytic virus inhibition of tumor cells [28]. This approach has demonstrated promising therapeutic effects; however, compared to membrane-bound vesicles, its manipulation is relatively less convenient. Meanwhile, strategies for surface modification of OVs with tumor cell membranes remain rare, and it remains unclear whether the effects of homologous and nonhomologous tumor cell membranes are the same.

In this study, we investigated the infection efficiency of OVs coated with tumor cell membranes from different sources on pancreatic cancer cells. We selected homologous tumor cell membranes derived from Panc02 pancreatic cancer cells and compared them with nonhomologous tumor cell membranes derived from B16-F10 melanoma cells. We found that the infection efficiency of the oncolytic virus coated with tumor cell membranes was improved, and compared to nonhomologous tumor cell membranes, Panc02 cell membrane-coated OVs had better infection performance. The virus titer in the tumor cells was significantly increased, effectively inhibiting tumor cell proliferation. Therefore, homologous tumor cell membranes have the potential to improve the low infection efficiency of OVs and solve current clinical treatment obstacles, improving the therapeutic effect of tumors. Our results suggest that tumor cell membrane-coated OVs may represent a promising approach for the treatment of tumors, addressing some of the current limitations in oncolytic virus therapy.

## 2. Materials and Methods

### 2.1. Materials and Cells

Panc02 pancreatic cancer cells and B16-F10 melanoma cells were obtained from Anhui Medical University and grown in Dulbecco's Modified Eagle's Medium supplemented with 10% fetal bovine serum (FBS) (Dalian Meilun Biotechnology Co., Ltd) and antibiotics. The oncolytic virus used in this study was obtained from the Chinese Academy of Sciences and modified to express a red fluorescent protein (RFP). Antibodies against cell markers were purchased from Dakewei Company and used according to the manufacturers' instructions. DAPI was purchased from Sigma–Aldrich. Dithiobis (succinimidyl propionate) (DSP), N-hydroxysuccinimide (NHS), and Igepal CA-630 were purchased from Sigma–Aldrich. All the solution and materials were used according to the instructions.

### 2.2. The Preparation of Tumor Cell Membranes-Coated OVs

Panc02 and B16-F10 cells were grown to 80% confluence in 10 cm dishes (NEST Biotechnology Co., Ltd). The cells were then washed with PBS and harvested using enzyme (Thermo Fisher Scientific). The cells were pelleted by centrifugation at 500× *g* for 5 min at 4°C. The cell pellets were resuspended in PBS containing DSP (5 mM) and NHS (50 mM) with the pipette (DLAB Scientific Co., Ltd) and incubated for 30 min at room temperature. The cross-linking reaction was stopped by adding glycine (250 mM) to the reaction mixture and incubating for 10 min at room temperature. The cell suspensions were then centrifuged at 500× *g* for 5 min at 4°C. The cell pellets were resuspended in cold lysis buffer (50 mM Tris-HCl, pH 7.4, 150 mM NaCl, 1% Igepal CA-630, protease inhibitors) and incubated on ice for 30 min. The lysates were centrifuged at 15,000× *g* for 30 min at 4°C to collect the membrane fractions. The membrane fractions were then resuspended in cold storage buffer (20% glycerol, 50 mM Tris-HCl, pH 7.4, protease inhibitors) and stored at −80°C until further use.

The oncolytic adenovirus was mixed with the tumor cell membranes using the PBS. Then, the mixed system was prepared by a liposome extruder into a membrane-coated oncolytic virus system. The coated viruses were then washed with PBS and resuspended in PBS for further experiments or storage at −80°C.

### 2.3. Infection of Tumor Cells with Oncolytic Virus

Panc02 cells were seeded in 24-well plates (SAINING Biotechnology Co., Ltd) at a density of 5 × 10^4^ cells per well. The following day, the cells were infected with the oncolytic virus of different formulations at a multiplicity of infection of 10 plaque-forming units per cell for 8 hr at 37°C. After infection, the cells were washed with phosphate-buffered saline (PBS) and cultured in a fresh medium.

### 2.4. Flow Cytometry Assays

Infected tumor cells were harvested by trypsinization, washed with PBS, and resuspended in Hank's Balanced Salt Solution containing 2% FBS. Finally, the cells were analyzed by flow cytometry using a BD LSR II instrument equipped with FACSDiva software (BD Biosciences). Data were analyzed using FlowJo software (Tree Star). The RFP in the tumor cells indicated the accumulation of homologous or nonhomologous tumor cell membrane-coated oncolytic virus.

### 2.5. In Vitro the Cell Viability

The proliferation of Panc02 cells was significantly affected by the treatment of homologous or nonhomologous tumor cell membrane coated oncolytic virus. Therefore, we incubated the pancreatic cancer cells with different formulations of oncolytic virus to determine the effect of antitumor cells. The cell viability of Panc02 cells was detected by the CCK8 kit (Cat#40203; Yeasen, Shanghai, China).

## 3. Results and Discussion

**Figure 1:**
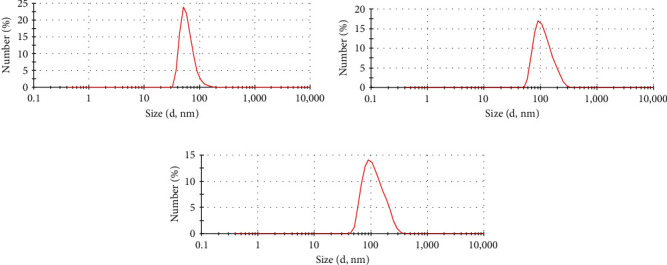
The diameter of tumor cell membrane-coated oncolytic viruses. The diameters of OVs (a), mB16-OVs (b), and m Panc02-OVs (c) measured by the Zetasizer Nano ZS.

The extracted Panc02 and B16 tumor cell membranes were mixed with the oncolytic virus at a certain ratio and then prepared into membrane-coated oncolytic virus systems using a liposome extruder. The prepared different systems were dissolved in PBS and their average sizes were detected using a dynamic light scattering instrument. The detection results showed that the unmodified oncolytic virus had a hydrodynamic diameter of approximately 91 nm (Figure 1(a)). After modification with B16 cell membranes, the diameter of the oncolytic virus was approximately 123 nm (Figure 1(b)), and after modification with PANC02 cell membranes, the diameter of the oncolytic virus was approximately 125 nm (Figure 1(c)). There was no significant difference in size between the two different cell membrane-modified OVs.

**Figure 2:**
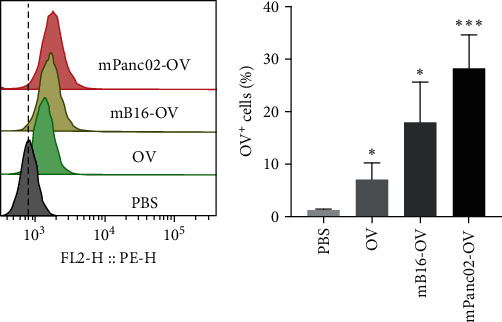
The infection ability of different formulations of oncolytic viruses in Panc02 cells was evaluated by flow cytometry.  ^*∗*^*p*  < 0.05,  ^*∗∗∗*^*p*  < 0.001.

Many OVs possess specific binding sites on their structures, which allow them to bind to receptors on the surface of human tumor cells and subsequently enter the cells. However, mouse tumor cells lack several receptors, making it difficult for OVs to effectively infect and enter these cells. The mechanisms underlying the differences in oncolytic virus infection between human and mouse tumor cells are not yet fully understood. However, it has been suggested that differences in cell surface receptor expression, intracellular signaling pathways, and immune responses may play a role in determining the susceptibility of these cells to oncolytic virus infection. Further studies are needed to elucidate these mechanisms and develop more effective oncolytic virus therapies for human cancer. To further verify whether the infection efficiency of OVs modified by homologous and nonhomologous tumor cell membranes is affected, we incubated different OVs with Panc02 cells. Since the oncolytic virus expresses RFP, it can be used as an indicator of virus titer inside tumor cells. We analyzed the intensity of red fluorescence in tumor cells after different treatments using flow cytometry. The experimental results in Figure 2 showed that the oncolytic virus without cell membrane encapsulation had a certain infection ability to tumor cells, and the oncolytic virus encapsulated by tumor cell membranes had a higher infection ability. Especially, the homologous tumor cell membrane-encapsulated oncolytic virus had the best infection effect, indicating that homologous tumor cell membrane is a potential strategy to improve the infection efficiency of OVs.

**Figure 3:**
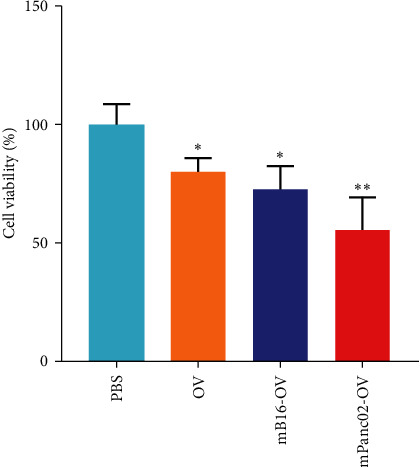
The cell viability in Panc02 cells triggered by the different formulations of oncolytic viruses.  ^*∗*^*p*  < 0.05,  ^*∗∗*^*p*  < 0.01.

After confirming the infection ability of different oncolytic virus systems, we further explored whether they could improve the killing effect on tumors. We incubated Panc02 tumor cells with different formulations of tumor cell membrane-modified OVs at 37°C for 24 hr, and then added CCK8 reagent to different samples. The absorbance of different sample wells was detected at 450 nm to evaluate their killing ability to tumor cells. The results in Figure 3 showed that infection with OVs could induce tumor cell death, and the killing ability was stronger with tumor cell membrane-modified OVs. Among them, homologous tumor cell membrane-encapsulated OVs had a more significant killing ability. These findings suggest that homologous tumor cell membrane, as a good modification system, can be used to solve the problem of low infection efficiency and poor treatment outcomes of OVs. Tumor cells often express specific proteins on their surfaces that are different from those on normal cells. When OVs are coated with the membrane of a tumor cell, they inherit these specific proteins. These proteins can interact with similar or complementary proteins on the surface of tumor cells in the body, facilitating the targeting and adherence of the nanoparticles to those cells. This can lead to receptor-mediated endocytosis, where the tumor cells internalize the OVs, allowing for the delivery of therapeutic agents directly inside the tumor cells.

## 4. Conclusion

Overall, OVs, as a highly potential tumor treatment strategy, are expected to solve the problem of poor treatment effect in current clinical tumor treatment. However, the low infectivity and induced body clearance mechanism of OVs seriously hinder the improvement of its effect. The existing tumor cell membrane coating strategy is an effective method to solve this problem, but whether the homologous tumor cell membrane is used to modify, or whether the nonhomologous tumor cell membrane can achieve the same effect, there are not many relevant studies to explore. Our research results show that the oncolytic virus wrapped in tumor cell membrane can improve the infection ability to tumor cells, but the homologous tumor cell membrane has a better effect. Therefore, the cell membrane derived from the same tumor cell is expected to become an excellent solution to improve the infection efficiency of tumor cells, thereby increasing its intracellular concentration and promoting the death of cancer cells. This study can greatly promote the clinical treatment plan based on OVs and improve the treatment effect of tumors.

## Data Availability

The data that support the findings of this study are available upon request from the corresponding author. The data are not publicly available due to privacy or ethical restrictions.

## References

[1] Minna J. D., Roth J. A., Gazdar A. F. (2002). Focus on pancreatic cancer. *Cancer Cell*.

[2] Schabath M. B., Cote M. L. (2019). Cancer progress and priorities: lung cancer. *Cancer Epidemiology, Biomarkers & Prevention*.

[3] de Groot P. M., Wu C. C., Carter B. W., Munden R. F. (2018). The epidemiology of lung cancer. *Translational Lung Cancer Research*.

[4] Travis W. D. (2002). Pathology of pancreatic cancer. *Clinics in Chest Medicine*.

[5] Chiocca E. A. (2002). Oncolytic viruses. *Nature Reviews Cancer*.

[6] Lawler S. E., Speranza M.-C., Cho C.-F., Chiocca E. A. (2017). Oncolytic viruses in cancer treatment. *JAMA Oncology*.

[7] Kaufman H. L., Kohlhapp F. J., Zloza A. (2015). Oncolytic viruses: a new class of immunotherapy drugs. *Nature Reviews Drug Discovery*.

[8] Vähä-Koskela M. J. V., Heikkilä J. E., Hinkkanen A. E. (2007). Oncolytic viruses in cancer therapy. *Cancer Letters*.

[9] Hemminki O., dos Santos J. M., Hemminki A. (2020). Oncolytic viruses for cancer immunotherapy. *Journal of Hematology & Oncology*.

[10] Hadac E. M., Peng K.-W., Nakamura T., Miller L. J., Russell S. J. (2004). Targeted measles virotherapy for pancreatic cancer. *Molecular Therapy*.

[11] Stevenson A. J., Giles M. S., Hall K. T. (2000). Specific oncolytic activity of herpesvirus saimiri in pancreatic cancer cells. *British Journal of Cancer*.

[12] Ady J. W., Heffner J., Klein E., Fong Y. (2014). Oncolytic viral therapy for pancreatic cancer: current research and future directions. *Oncolytic Virotherapy*.

[13] Al Caghchi C., Zhang Z., Alusi G., Lemoine N. R., Wang Y. (2015). Vaccinia virus, a promising new therapeutic agent for pancreatic cancer. *Immunotherapy*.

[14] Power A. T., Bell J. C. (2007). Cell-based delivery of oncolytic viruses: a new strategic alliance for a biological strike against cancer. *Molecular Therapy*.

[15] Howard F., Muthana M. (2020). Designer nanocarriers for navigating the systemic delivery of oncolytic viruses. *Nanomedicine*.

[16] Yokoda R., Nagalo B. M., Vernon B. (2017). Oncolytic virus delivery: from nano-pharmacodynamics to enhanced oncolytic effect. *Oncolytic Virotherapy*.

[17] Nakashima H., Kaur B., Chiocca E. A. (2010). Directing systemic oncolytic viral delivery to tumors via carrier cells. *Cytokine & Growth Factor Reviews*.

[18] Hill C., Carlisle R. (2019). Achieving systemic delivery of oncolytic viruses. *Expert Opinion on Drug Delivery*.

[19] Ferguson M. S., Lemoine N. R., Wang Y. (2012). Systemic delivery of oncolytic viruses: hopes and hurdles. *Advances in Virology*.

[20] Atasheva S., Shayakhmetov D. M. (2021). Oncolytic viruses for systemic administration: engineering a whole different animal. *Molecular Therapy*.

[21] Cook M., Chauhan A. (2020). Clinical application of oncolytic viruses: a systematic review. *International Journal of Molecular Sciences*.

[22] Santos J., Heiniö C., Quixabeira D. (2021). Systemic delivery of oncolytic adenovirus to tumors using tumor-infiltrating lymphocytes as carriers. *Cells*.

[23] Li R., He Y., Zhang S., Qin J., Wang J. (2018). Cell membrane-based nanoparticles: a new biomimetic platform for tumor diagnosis and treatment. *Acta Pharmaceutica Sinica B*.

[24] Harris J. C., Scully M. A., Day E. S. (2019). Cancer cell membrane-coated nanoparticles for cancer management. *Cancers*.

[25] Rao L., Yu G.-T., Meng Q.-F. (2019). Cancer cell membrane-coated nanoparticles for personalized therapy in patient-derived xenograft models. *Advanced Functional Materials*.

[26] Oroojalian F., Beygi M., Baradaran B., Mokhtarzadeh A., Shahbazi M-Ali (2021). Immune cell membrane-coated biomimetic nanoparticles for targeted cancer therapy. *Small*.

[27] Thakur A., Parra D. C., Motallebnejad P., Brocchi M., Chen H. J. (2022). Exosomes: small vesicles with big roles in cancer, vaccine development, and therapeutics. *Bioactive Materials*.

[28] Wedge M.-E., Jennings V. A., Crupi M. J. F. (2022). Virally programmed extracellular vesicles sensitize cancer cells to oncolytic virus and small molecule therapy. *Nature Communications*.

